# Subarachnoid neurocysticercosis in a rural area of the Bolivian Chaco: A population-based survey through the use of a novel urine antigen assay

**DOI:** 10.1371/journal.pntd.0014214

**Published:** 2026-04-27

**Authors:** Anna Barbiero, Maria Micieli, Yesenia Castillo, Cindy Espinoza Guerrero, Tiziana Di Maggio, Valentina Petrini, Michele Spinicci, Chiara Aguzzoli, Bianca Ribelli, Fabio Macchioni, Simona Gabrielli, Silvio Menacho, Marianne Strohmeyer, Mimmo Roselli, Veronica Poma, Reinelda Cuellar, Elizabeth Crespo, Joaquín Monasterio, Francesco Cosmi, Walter Mario Camargo, Seth O’Neal, Hector Hugo Garcia, Alessandra Nicoletti, Alessandro Bartoloni

**Affiliations:** 1 Department of Experimental and Clinical Medicine, University of Florence, Florence, Italy; 2 Center for Global Health and Research and Development Laboratories, Universidad Peruana Cayetano Heredia, Lima, Peru; 3 Department of Medical Biotechnologies, University of Siena, Siena, Italy; 4 Infectious and Tropical Diseases Unit, Careggi University Hospital, Florence, Italy; 5 School of Human Health Sciences, University of Florence, Florence, Italy; 6 Department of Veterinary Sciences, University of Pisa, Pisa, Italy; 7 Department of Public Health and Infectious Diseases, Sapienza University of Rome, Rome, Italy; 8 Eiti Health Center, Eiti, Gutierrez, Bolivia; 9 Escuela de Salud del Chaco Tekove Katu, Gutierrez, Bolivia; 10 Convenio de Salud, Camiri, Bolivia; 11 Hospital Hernández Vera, Santa Cruz, Bolivia; 12 Servicio Departamental de Salud (SEDES) de Santa Cruz, Santa Cruz, Bolivia; 13 Medical Center Neuro Center, Santa Cruz, Bolivia; 14 School of Public Health, Oregon Health and Science University - Portland State University, Portland, United States of America; 15 Cysticercosis Unit, Instituto Nacional de Ciencias Neurológicas, Lima, Peru; 16 Department of Medical, Surgical Sciences and Advanced Technologies “G.F. Ingrassia” (DGFI), University of Catania, Catania, Italy; George Washington University School of Medicine and Health Sciences, UNITED STATES OF AMERICA

## Abstract

Neurocysticercosis (NCC) is a major public health concern in low-middle-income countries. Subarachnoid NCC (SANCC), a less frequent form of NCC, is characterized by severe clinical evolution, high mortality and impactful neurological sequelae in survivors. However, due to a long pre-clinical period, early diagnosis and treatment of SANCC would likely improve the clinical course and reduce the occurrence of complications. To date, data on the prevalence of SANCC in endemic settings are scant, and there are no commercially available tests for SANCC screening in at-risk populations. Between October 2023 and May 2024, we conducted a population-based cross-sectional study aimed at implementing a non-invasive screening for SANCC in a low-resource area of the Plurinational State of Bolivia, using a home-made Ag-ELISA urine assay based on the monoclonal antibody set TsW5-TsW8. Questionnaires were also administered to the study population to assess the presence of behaviors and risk factors for active transmission of *Taenia solium* in the area. The study involved 1,232 subjects aged ≥10 years, 1,130 of whom delivered a urine sample. Among them, a median age of 27.4 years was observed, with 38.1% of subjects being males. Overall, 7 subjects with positive urine antigen test, confirmed on two different samples, were identified and underwent magnetic resonance imaging. The diagnosis of SANCC was radiologically confirmed in 4/7 subjects, for a prevalence of SANCC in the enrolled population of 3.54/1,000. The antigen assay showed a positive predictive value for SANCC of 57.1%. Risk factors for active transmission of *T. solium*, such as lack of access to adequate sanitation and the widespread practice of domestic pig breeding and sacrifice, were frequent. While further studies are needed to better define the diagnostic performance of the employed urine assay, this study confirms the presence of SANCC in the study area, consistent with evidence from similar endemic settings.

## Author summary

Human neurocysticercosis (NCC) is a parasitic disease caused by the localization of the larval stage of *Taenia solium* in the central nervous system after ingestion of the parasitic eggs. It represents a relevant health issue in low-middle-income countries. Subarachnoid NCC (SANCC) is an uncommon but severe form of NCC, in which parasites grow uncontrolledly within the subarachnoid space, causing increased intracranial pressure, thus determining a slowly progressive, often fatal disease. Early diagnosis and treatment of SANCC could improve its clinical course. In this study, we used a recently developed urine-based assay to screen for SANCC in a rural area of the Bolivian Chaco, where NCC accounts for nearly 30% of epilepsy cases, and risk factors for the parasite transmission are widespread. We identified a non-negligible number of SANCC cases confirmed by magnetic resonance imaging, with a prevalence of 3.54 per 1,000 individuals. Notably, 57.1% of subjects with a positive urine test had radiological confirmation of SANCC, suggesting a good positive predictive value of the assay. These findings highlight the ongoing public health impact of NCC in the study area and support further research to establish the diagnostic performances and implementation feasibility of the employed urine assay as a screening tool.

## Introduction

Taeniasis and cysticercosis are major public health issues worldwide. In endemic regions, including Latin America, neurocysticercosis (NCC) is a common cause of neurological morbidity and a leading cause of epilepsy [1]. NCC is caused by the infection of the central nervous system with the larval stage of *Taenia solium*, with clinical manifestations depending on the number of lesions, their location, as well as the intensity of the immunological reaction against the parasites.

In the rural areas of the Bolivian Chaco (a semiarid region of the Plurinational State of Bolivia, located in the South-East area of the country), a study carried out in 1994 showed that 27.4% of people with epilepsy (PWE) met Del Brutto’s diagnostic criteria for definite or probable NCC [2]. This finding was also confirmed by a more recent study carried out in the same area, where a prevalence of NCC among PWE was 22.7% [3].

NCC is associated with high morbidity and mortality, as well as stigmatization and low quality of life [4]. In parenchymal NCC, the most frequent symptoms are seizures, that may persist for years, even after the parasite has calcified. Extra-parenchymal NCC (i.e., lesions within the subarachnoid space or ventricular system) is rarer than parenchymal NCC, but often more severe. The most common symptoms are headache in its early stages and fatal forms of intracranial hypertension in the advanced disease. When cysts are present in the subarachnoid space (subarachnoid NCC, SANCC) uncontrolled lesion growth can cause a mass effect, resulting in elevated intracranial pressure and hydrocephalus, thus leading to a progressive and often fatal form of disease [5,6]. SANCC is characterized by a long incubation period (10–25 years) and an insidious disease onset, with symptoms often occurring only at advanced stages of the disease, when treatment options are limited and complex surgical management is often required [7]. For this reason, early detection, defined as the identification of SANCC before the onset of severe clinical manifestations such as hydrocephalus or intracranial hypertension, could improve outcomes and facilitate easier management in affected subjects. Currently, no validated and commercially available screening tests for SANCC are available; hence, data regarding the prevalence and health burden of SANCC in endemic areas are very scarce [8].

Since cysticercosis antigens tend to be detected at higher levels in case of SANCC [9–11], antigen (Ag) based urine tests have recently been developed to predict the risk of SANCC vs parenchymal NCC. This is likely because subarachnoid cysts grow freely within cerebrospinal fluid spaces and remain viable for prolonged periods, resulting in increased release of circulating parasite antigens.

These assays represent a promising, non-invasive and accessible screening tool for the early identification of SANCC in low-resource settings, where taeniasis and cysticercosis are endemic. A study conducted by McCleery et al. in 2020 in rural communities of northern Peru, showed that an in-house Ag-ELISA urine assay for the detection of cysticercosis Ag, based on monoclonal antibody set B158/B60, had a positive predictive value (PPV) of 62% for SANCC and 77% for any type of NCC, highlighting the possible role of this test in the early detection of SANCC [12].

Another novel Ag-ELISA, based on the monoclonal antibody TsW8 as the capture antibody and TsW5 as the detection antibody, showed the ability to detect circulating cyst antigens in urine samples of patients with viable NCC infections [13]; for the same test, high Ag measurement correlations with the widely used B158/B60 Ag-ELISA on serum sample across diverse types of NCC were reported [14]. No data are currently available in the literature on the diagnostic performance of the TsW8-TsW5 urine Ag-ELISA for SANCC screening in endemic settings.

In the present research, we report the results of a pilot community-based cross-sectional study in which SANCC screening was performed with the TsW8-TsW5 Ag-ELISA assay, in four rural communities of the Bolivian Chaco (Plurinational State of Bolivia).

## Materials and methods

### Ethics statement

The study was approved by the local ethics committee “Colegio Médico de Santa Cruz” (registration code: TDEDM CMSC-029/2023). Enrolled patients provided written informed consent for study participation, and each record was pseudonymized before compilation of the Case Report Form (CRF). For subjects younger than 18 years, written informed consent was obatined from parents or legal guardians. The study was conducted in accordance with the ethical principles of the Declaration of Helsinki and with the Good Clinical Practice guidelines.

The study was conducted in collaboration with the Convenio de Salud – Vicariato de Camiri, and the Escuela de Salud del Chaco, Tekove Katu, Gutierrez.

### Study area and population

**Fig 1:**
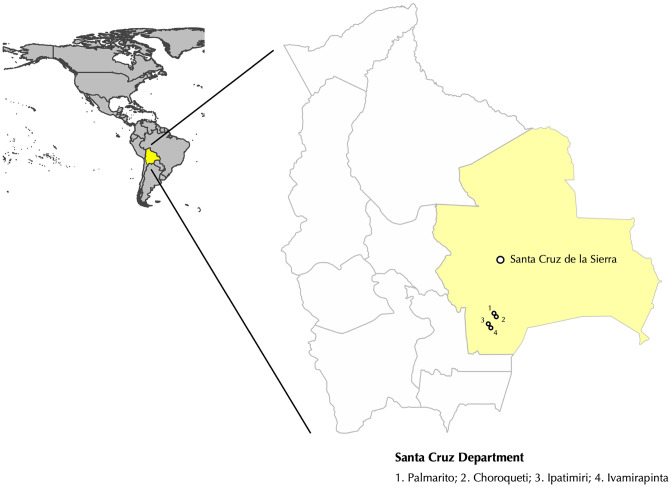
Geographic representation of the four involved rural communities (1. Palmarito; 2. Choroqueti; 3. Ipatimiri; 4. Ivamirapinta) located in the Santa Cruz Department, Plurinational State of Bolivia. Base map showing national and administrative boundaries derived from the Natural Earth dataset (https://www.naturalearthdata.com/downloads/10m-cultural-vectors/). Natural Earth data are in the public domain; license information is available at https://www.naturalearthdata.com/about/terms-of-use/. The map was generated using QGIS.

The study area included four rural communities of the Bolivian Chaco, Cordillera Province, Santa Cruz Department: Ivamirapinta, Ipatimiri, Choroqueti and Palmarito (Fig 1). According to available census data (2023) provided by local health authorities (Convenio de Salud, Camiri), the overall population in the study area is 3,130 inhabitants (1,178 in Ivamirapinta, 610 in Palmarito, 229 in Choroqueti and 1,113 in Ipatimiri), with 2,313 (73.9%) older than 10 years.

### Sample size and inclusion criteria

The study included all individulas residing in the study area aged ≥ 10 years and giving written informed consent to participate. Individuals aged ≥ 10 years were included to increase the likelihood of detecting clinically relevant infections, given the long incubation period of SANCC.

Based on the total population of individuals aged ≥ 10 years across the four rural communities, and assuming a participation rate of 80%, and a predicted prevalence of SANCC of 0.8% [12], we calculated a sample size of 1,000 subjects in order to achieve a 95% confidence level with a margin of error of ± 0.5%, using a single-proportion sample size formula to estimate prevalence with absolute precision.

### Study design and sampling

A community-based cross-sectional study was conducted during the period October 2023-May 2024.

In each community, the study design and objectives were presented to local authorities and health personnel. The study purpose was then shared with the community through educational and interactive meetings. In the following days, the investigative team conducted a door-to-door survey, during which all individuals who consented to participate to the study were given a questionnaire and were provided with a plastic urine container. Participants were asked to provide a urine sample either immediately upon enrollment or within the following two days at the village health center.

### Questionnaire survey

Two different questionnaires were used for the population survey. The first questionnaire, designed to gather information on households characteristics, including the number of family members, toilet availability, the presence of pigs (either owned or nearby), and the number of owned pigs, was provided to each surveyed family. The second questionnaire, which collected individual data on demographics, occupation, education level and habit of outdoor defecation, was provided to each enrolled participant.

### Sample collection and laboratory analysis

Urine samples collected during the period October-December 2023 were aliquoted in 2 mL vials, preserved at room temperature at the local health facility in Camiri (Convenio de Salud) and shipped to the Parasitic Immunology Laboratory at the Universidad Peruana Cayetano Heredia, Lima, Peru, within one month after sample collection. Samples were analyzed for cysticercosis Ag using ELISA (Ag-ELISA) based on monoclonal antibody set TsW5-TsW8, as previously described [13]. Results were expressed as a ratio of the optical density (OD) of the sample divided by the mean OD of eight known negative samples plus 3 standard deviations (SDs).

In May 2024, a second urine sample was collected from partecipants with a previous positive result. Samples were preserved at -20° C within one hour after collection and stored frozen until processed. Urine samples were analysed by performing the same Ag-ELISA test used in the first phase.

### Neuroimaging and SANCC diagnosis

Brain magnetic resonance imaging (MRI) was used as a confirmatory diagnostic method among individuals with confirmed antigen positivity. Non-contrast MRI was offered to participants with a urine OD ratio ≥3 confirmed on the second urine sample; the cut-off of an OD ratio ≥3 was established, based on previous experiences, to reduce false-positive results and maximize the positive predictive value (PPV) of the screening test [12].

Brain MRI was conducted using a 1.5-tesla machine with T2, FLAIR, and TrueFISP sequences.

Participants with confirmed NCC at the MRI were referred to a local neurologist for management and treatment. MRIs were performed and interpreted at the Medical Center Neuro Center, Santa Cruz, Plurinational State of Bolivia. Diagnosis of SANCC was made according to the revised diagnostic criteria for neurocysticercosis [15].

### Data collection and statistical analysis

Information collected for each participant through family and individual questionnaires, urine assay and MRI results were recorded using REDCap 8.11.6. (Project REDCap, USA).

Continuous variables were described using medians and inter-quartile ranges (IQR), whereas categorical variables were analysed as frequencies and proportions. Differences between categorical variables were evaluated with Chi-square test or Fisher’s exact test, as appropriate, and continuous variables with Wilcoxon rank-sum test.

Point-prevalence was calculated based on the number of patients affected by SANCC living in the study area who fulfilled the revised diagnostic criteria for NCC (Del Brutto 2017) on prevalence day (01.11.2023). Confidence intervals (CIs) for estimates were calculated assuming a Poisson distribution.

The PPV of the urine assay was calculated as the proportion between radiologically and clinically confirmed SANCC cases and the number of positive urine assays (VP/VP + FP).

Results were considered statistically significant for *p*-values < 0.05. Statistical analyses were performed using STATA v18.0 (STATACorp, USA).

## Results

### Baseline individual characteristcs

Overall, 1,232 subjects living in the study area were contacted and interviewed, of which 1,130 provided a urine sample (participation rate 91.7%). Among those who provided the urine sample, 431 (38.1%) were men, and the median age was 27.4 [IQR: 15.7-48.2]. Considering the baseline characteristics of the 102 subjects who were not screened, the majority were male (60, 58.8%) and significantly younger (mean age of 28.4 ± 15.6) with respect to the screened subjects.

Baseline characteristics and information collected through the individual interviews of the enrolled population, divided by community of residence, are reported in Table 1.

**Table 1:** Distribution of demographics and information collected through individual interviews, divided by community of residence.

	Ivamirapinta (n = 318)	Ipatimiri (n = 382)	Choroqueti (n = 127)	Palmarito (n = 303)	*Total (n = 1,130)*
**Male sex**	119 (37.4)	144 (37.7)	48 (37.8)	120 (39.6)	431 (38.1)
**Median age [IQR]**	26.7 [15.0-49.5]	28.5 [15.4 – 50.3]	25.7 [16.5-45.7]	28.2 [16.2-46.2]	27.4 [15.7-48.2]
**Education level**					
None	16/305 (5.3)	12/367 (3.3)	5/127 (3.9)	14/295 (4.8)	47/1,094 (4.2)
Primary-school	120/305 (39.3)	123/367 (33.5)	61/127 (48.0)	114/295 (38.6)	418/1,094 (38.2)
Secondary-school	110/305 (36.1)	144/367 (39.2)	42/127 (33.1)	104/295 (35.3)	400/1,094 (36.6)
High-school	51/305 (16.7)	68/367 (18.5)	17/127 (13.4)	47/295 (15.9)	183/1,094 (16.7)
Professional course	5/305 (1.6)	4/367 (1.1)	1/127 (0.8)	6/295 (2.0)	16/1,094 (1.5)
College	3/305 (1.0)	16/367 (4.4)	1/127 (0.8)	10/295 (3.4)	30/1,094 (2.7)
**Most common defecation place**					
WC	16/314 (5.1)	32/374 (8.6)	25/127 (19.7)	18/296 (6.1)	91/1,111 (8.2)
Latrine	237/314 (75.5)	303/374 (81.0)	99/127 (78.0)	200/296 (67.6)	839/1,111 (75.5)
Open field	61/314 (19.4)	39/374 (10.4)	3/127 (2.4)	78/296 (26.4)	181/1,111 (16.3)
**Toilet availability**					
None	50/313 (16.0)	30/379 (7.9)	2/127 (1.6)	78/303 (25.7)	160/1,122 (14.3)
Latrine	247/313 (78.9)	318/379 (83.9)	103/127 (81.1)	207/303 (68.3)	875/1,122 (78.0)
WC	16/313 (5.1)	31/379 (8.2)	22/127 (17.3)	18/303 (5.9)	87/1,122 (7.8)
**Eating meat with visible cysts**					
Yes	12/281 (4.3)	8/362 (2.2)	4/114 (3.5)	13/255 (5.1)	37/1,012 (3.7)
No	269/281 (95.7)	354/362 (97.8)	110/114 (96.5)	242/255 (94.9)	975/1,012 (96.3)
**Knowledge of disease transmission**					
Yes	216/315 (68.6)	338/372 (90.9)	82/127 (64.6)	247/296 (83.5)	883/1,110 (79.6)
No	99/315 (31.4)	34/372 (9.1)	45/127 (35.4)	49/296 (16.6)	227/1,110 (20.5)
**Owning pigs**					
Yes	164/313 (52.4)	162/374 (43.3)	71/127 (55.9)	133/303 (43.9)	530/1,117 (47.5)
No	149/313 (47.6)	212/374 (56.7)	56/127 (44.1)	170/303 (56.1)	587/1,117 (52.6)
**Presence of pigs around the household**					
Yes	281/311 (90.4)	328/371 (88.4)	116/127 (91.3)	275/295 (93.2)	1000/1,104 (90.6)
No	30/311 (9.7)	43/371 (11.6)	11/127 (8.7)	20/295 (6.8)	104/1,104 (9.4)

### Household characteristics

The survey included 367 households and reported a median of 5 members per household (IQR: 3–7), with a median of 3 members aged ≥ 10 (IQR: 2 – 5). Concerning toilet availability, presence of a WC was reported in 28/365 (7.7%) of the households, whereas a latrine was present in 288/365 (79.0%) and absence of any toilet facility was reported in 49/365 households (13.4%). Toilet availability was similar across the selected communities, except for Choroqueti where the presence of a WC was significantly more common (6/32 households, 18.8%) compared to the other communities (8/105, 7.6% in Ivamirapinta; 9/135, 6.7% in Ipatimiri; 5/93, 5.4% in Palmarito; *p*-value = 0.017).

One-hundred-fifty out of 367 (40.9%) households owned pigs, with a median of 4 pigs per household (IQR: 2 – 7). Of these, 37 (24.7%) declared that their pigs are never enclosed and 26 (17.3%) of the 150 pig owners reported history of cysticercosis in their pigs. Domestical slaughter of property pigs was reported by 114/150 (76.0%) pig owners. The presence of free-roaming pigs around the house was reported in 214/367 (58.3%) households. Overall, no significant differences in the frequency of pig ownership, presence of pigs around the house, pig confinement, history of porcine cysticercosis and domestic slaughter of property pigs were observed among the four involved communities.

### Urine assays

Of the 1,130 samples collected in the period October-December 2023, which were preserved at room temperature and sent to the Parasitic Immunology Laboratory at the Universidad Peruana Cayetano Heredia, 86 (7.6%) tested positive (i.e., with an OD ratio ≥3) in the Ag-ELISA assay, with a median OD ratio of 21.0 (IQR: 7.6 – 38.8). No significant differences in the distribution of positive cases among sexes nor across the four involved rural communities were observed. Given the unexpectedly high prevalence of positive assays and the suspicion of high false-positive rates, a second urine sample was collected from 76/86 subjects who initially tested positive.

To address the concern that room temperature preservation (25°-35° C) might have lowered the assay specificity, the newly collected urine samples were preserved and stored at -20° within one hour after collection until laboratory analysis was performed. Of the 76 newly collected samples, the OD ratio was confirmed to be ≥ 3 in 7 cases (median OD ratio 10.2, IQR: 5.1 – 17.1). Overall, among the tested population, 0.6% (7/1,130) had a confirmed positive urine cysticercosis Ag screening.

### Brain imaging and SANCC

**Fig 2:**
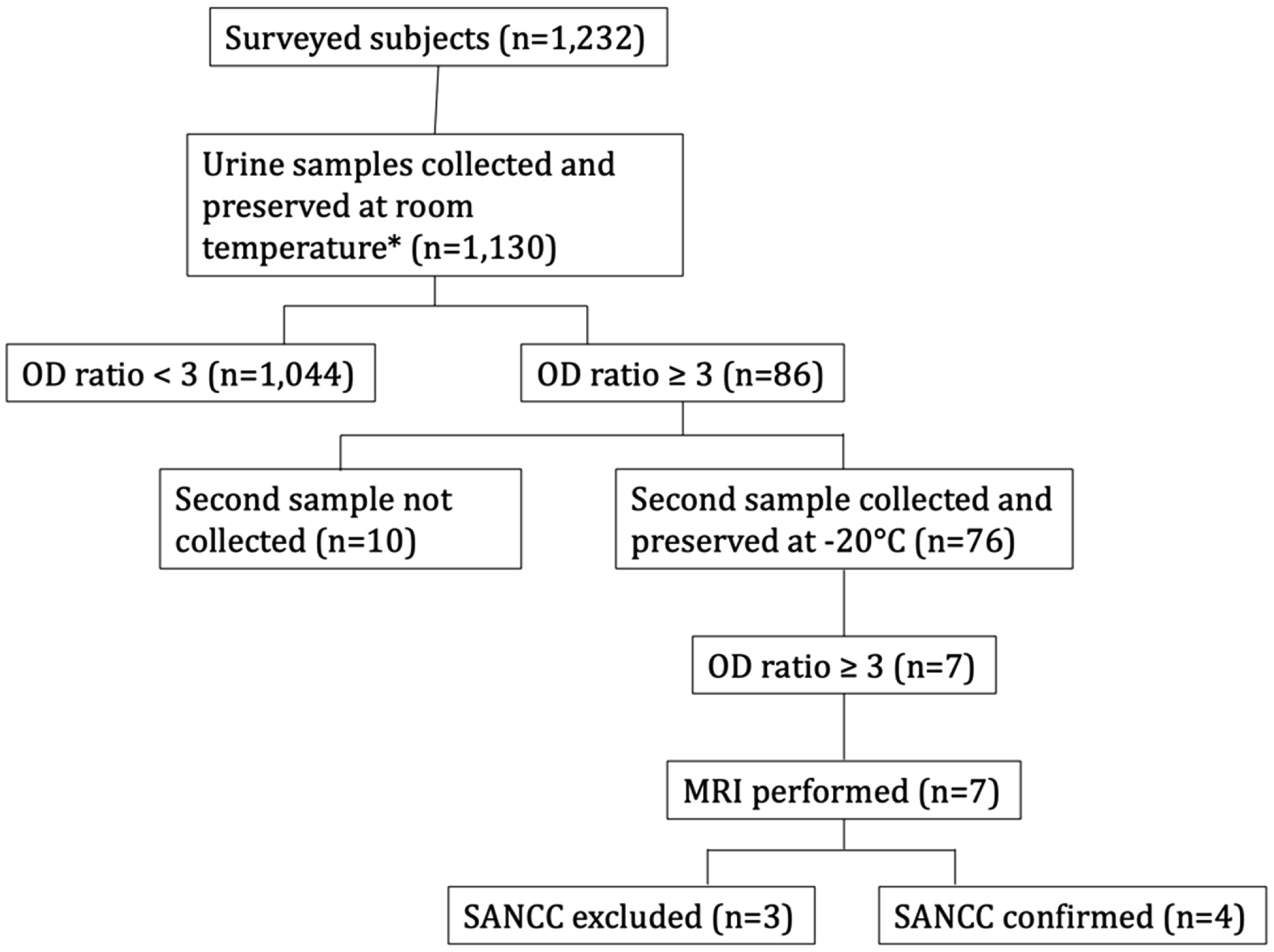
Study flow showing the main study results from the population screening for cysticercosis antigens in urine to detect SANCC. OD ratio = optical density for antigen capture using ELISA based on monoclonal antibody set TsW5-TsW8. MRI = magnetic resonance image; NCC = neurocysticercosis. *Analysis performed within one month after collection.

**Fig 3:**
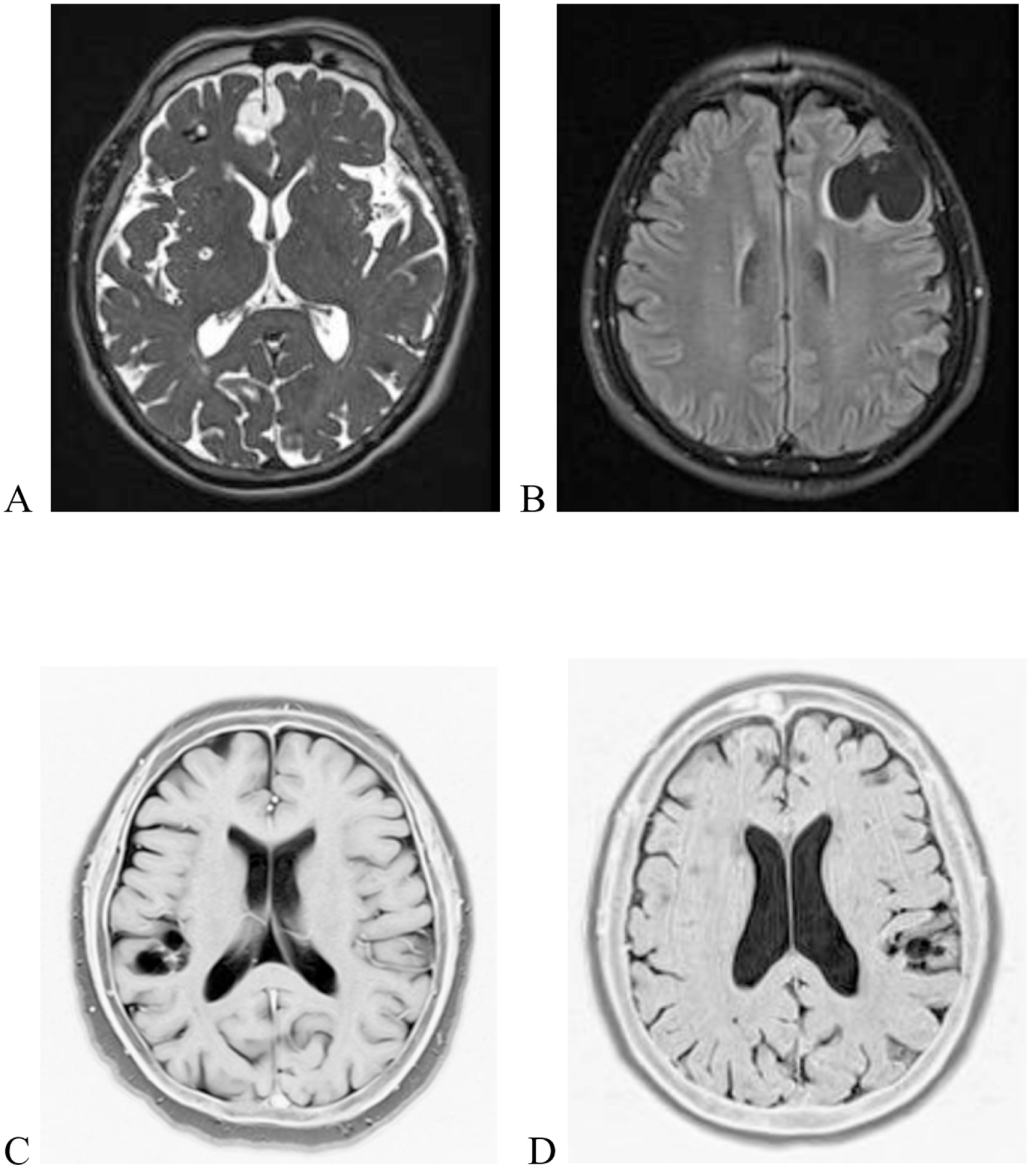
Representative images of brain MRI findings in subjects with radiologically confirmed SANCC. A. Multiple intra-axial and extra-axial viable cysticerci in vesicular stage (subject 2), T2-weighted turbo spin-echo sequence; B. Viable cysticerci occupying the left frontal region with an extra-axial location (subject 1), T2-weighted STIR dark-fluid sequence; C. Viable cysticerci occupying the right parietotemporal lobe, with an extra-axial location (subject 4), T2-wighted turbo spin-echo sequence (grayscale inverted); D. Viable cysticerci clusterized in the parietal left parietal sulcus (subject 3), T2-weighted SPACE sequence (grayscale inverted).

The seven subjects who were confirmed positive at both the first and second urine Ag-ELISA assay underwent brain MRI. NCC was documented in 4 cases (two men and two women). The study flow is represented in Fig 2. All MRI-positive cases presented SANCC (Fig 3), leading to a prevalence of 3.54/1,000 (95% CI 1.00-9.04). All cases were aged ≥ 54, with a median age (62.3 years; IQR: 57.5-69.3) significantly higher (*p*-value = 0.0076) than that of the population without confirmed SANCC (27.3 years; IQR: 15.6-48.1). The low number of cases did not allow analysis of any correlations between SANCC cases and other individual or household risk factors.

The PPV for SANCC of the urine Ag assay was 57.1% (4/7; 95% CI 50.4 – 90.1) at an OD ratio ≥ 3. Notably, most SANCC cases were asymptomatic or reported only mild, non-specific symptoms at the time of diagnosis: three cases reported a positive history of headache, while one (subject 2) was affected by epilepsy. The main clinical, demographic and radiological characteristics of the subjects with a confirmed urine OD ratio ≥ 3 are reported in Table 2.

**Table 2:** Main clinical and demographical characteristics of subjects with confirmed positive Ag-ELISA (OD ratio ≥3) on urine samples.

Subjects	Sex	Age	Community	OD ratio	Symptoms	MRI result
1	M	54	Palmarito	5.370	Headache	Parenchymal NCC + SANCC
2	M	64	Palmarito	36.270	Epilepsy	Parenchymal NCC + SANCC
3	F	73	Ipatimiri	10.986	Headache, dizziness	Parenchymal NCC + SANCC
4	F	59	Ipatimiri	17.151	Headache	Parenchymal NCC + SANCC
5	F	16	Palmarito	5.110	Asymptomatic	Negative
6	M	75	Ivamirapinta	10.169	Asymptomatic	Negative
7	M	11	Ipatimiri	3.930	Asymptomatic	Negative

Of note, MRI was also offered to the 26 subjects who tested positive during the first phase with an OD ratio ≥ 30, but who tested negative at the second urine sample collection. MRI resulted negative for NCC/SANCC in all of them, confirming the suspicion of high false-positive rate in the urine samples stored at room temperature before the analysis.

## Discussion

Bolivia is a middle-income country where almost 4 million people live under the “poverty line” and where access to the health system is still difficult, especially in rural areas [16]. The southeast region of Bolivia is part of the Gran Chaco, a subtropical area also including Argentina and Paraguay, characterized by a low population density. The ethnic group living in the study area is mainly represented by native Guaraní people, living in poor dwellings located in rural communities often reachable only by rural roads, without running water or electricity, and with a local economy based on agriculture and animal husbandry. These socio-environmental conditions provide a plausible context for the persistence of the sanitation- and livestock-related risk factors for *T. solium* transmission.

In this study, we used a recently developed in-house Ag-ELISA urine assay for the detection of cysticercosis Ag, based on monoclonal antibody set TsW5-TsW8 [8,13,14,17], to conduct a pilot cross-sectional screening study for SANCC in some rural communities of the Bolivian Chaco. Moreover, we surveyed through interviews with the enrolled population, the presence of behaviors and risk factors related to the life-cycle perpetuation of *T. solium.*

The high participation rate to this study (91.7%), partly explained by the long-lasting collaboration between the involved Italian and Bolivian research teams and the strong trust-based relationship with local population [18], also confirms the high acceptability of the implemented urine assay as a screening test.

Regarding the information collected through the interviews, despite a high level of awareness regarding disease transmission and prevention strategies, the collected data confirmed the widespread presence of risk factors for *T. solium* transmission, such as the frequent practice of open-field defecation (reported by 16.3% of the interviewed subjects) [19]. Of note, latrines were reported as the most commonly used hygenic facility (78.0%). While latrines can represent an accessible and feasible solution to limit enviromental dispersion of potentially contaminated faeces, their effectiveness depends on correct management and on their inaccessibility to roaming pigs to guarantee effective control of *T. solium* transmission. In the study area, the widely reported presence of free-roaming pigs, combined with the accessibility to latrines that are not properly enclosed, likely represents one of the main sources of infection for pigs, with latrines ending up acting as a hotspot, rather than a containment, for porcine cysticercosis [20,21].

Regarding the screening results, urine antigen assay and MRI led to the detection of four cases of SANCC, with a prevalence of 3.54/1,000 in the study population. Our result is comparable, although slightly lower, to the prevalence (0.8%) observed in the study conducted by McCleery et al., conducted in Northern Peru with a similar methodology but with the use of the monoclonal antibody set B158/B60 for SANCC screening [12]. To the best of our knowledge, further data on the overall prevalence of SANCC in endemic areas are not available at the time of writing this report [6]. Of note, the use of urine antigen as a detecting tool for SANCC is based on very high antigen levels in individuals with SANCC and thus it does not allow to estimate the burden of individuals with parenchymal NCC, particularly those with few cysts who usually present low or even negative antigen levels. Considering that SANCC is presumed to represent a minority, although not negligible, of all NCC cases [6], our results suggest a likely relevant burden of NCC in the study area, as supported by previous studies conducted on NCC and PWE in the same setting [2,3]. The presence of NCC or SANCC cases in a specific area is not necessarily related to an ongoing active *T. solium* life cycle, since these cases could be related to infections acquired many years earlier. On the other hand, although surveillance programs aimed at defining the active transmission of *T. solium* were not implemented at the time of the study, local healthcare workers report that some cases of taeniasis still occur in the study area, suggesting that *T. solium* circulation could be currently active.

While the low number of confirmed cases of SANCC does not allow to analyze correlations with demographic and risk factors characteristic in the affected subjects, the older age observed in those with SANCC compared to the general study population is in line with previous literature data [22].

According to the number of cases with confirmed positivity on two urine collected samples, the urine antigen assay showed a PPV of 57.1%, slightly inferior but comparable with the results reported in the study conducted by McCleery et al. (PPV 62.0%), where the urine assay based on the monoclonal antibody set B158/B60 was used [12]. Of note, there is no reason to expect a differential lack of specificity between the TsW5-TsW8 and B158/B60 assay, since the former is basically the same assay but using homologous monoclonal antibodies produced against *T. solium* rather than *T. saginata* [13] and has consistently proven reliable and robust [17,23].

A crucial element of this study was the observed difference in the number of positive urine assays and their OD ratios between the first (86 positive samples with a median OD ratio of 21.0) and the second (7 positive samples with median OD ratio of 10.2) collection, that were performed four months apart and differentiated only by a significant period of sample preservation at room temperature in a tropical area. Indeed, although only one sample collection was initially planned, the unexpectedly high number of positive cases during the first collection suggested that some confounding factor could have altered the specificity of the test, leading to an excessive number of false-positive results. Since samples were preserved and transported at room temperature (25°-35° C) during the first phase, differently from previous studies in which samples were preserved at -20° C [12], the second collection was made maintaining the samples temperature at -20° C until laboratory analysis. Moreover, 26 subjects with a highly positive first sample but with a negative result at the second collection underwent MRI, which resulted negative for all of them, confirming the suspicion of high false positive results rates during the first collection phase. Of note, no specific antiparasitic treatments were administered nor were massive drug administration programs implemented between the two urine sample collections. These observations support the hypothesis that preservation temperature could have had a crucial role in altering test specificity. False-positive results caused by cross-reactions with other parasitic infections or presence of *T. solium* cysts outside the central nervous system cannot be excluded but alone would not explain the relevant differences observed between the two collections.

Mechanisms explaining the relationship between preservation temperature and test specificity are not known, and work is in progress with other samples to confirm test reproducibility and effects of sample storage conditions on its diagnostic and screening performances. The feasibility and applicability of this test will therefore need to be further assessed considering average temperatures in different endemic areas and the possible need for appropriate cold chain maintenance for its implementation. In this perspective, it is worth mentioning that the TsW8-TsW5 assay has also been described as a point-of-care (POC) urine lateral flow test, potentially representing a very attractive alternative in low-resource settings [17]. Unfortunately, it was not available by the time of our study to be used in the field before the urine samples were transported or repeated in parallel with the ELISA format.

An important limitation of this study lays in the fact that MRI were not performed in a negative control group, therefore test sensitivity and specificity could not be determined.

In conclusion, the urine antigen assay employed in this study for SANCC screening allowed the detection of 4 cases of SANCC, the prevalence of which resulted to be compatible with epidemiological data coming from other *T. solium* endemic areas; however, further research is needed to better define its diagnostic performances and applicability in limited resource settings. Currently, a study in northern Peru including about 30,000 urine samples is assessing the predictive value of the TsW8/TsW5 Ag-ELISA to screen for SANCC [17]. This study confirms not only the presence of widespread risk factors for the perpetuation of *T. solium* transmission, but also the presence of a low, but relevant, number of cases of SANCC in the study area. Considering that this is a less common form of NCC as well as the severe neurological morbidity associated with SANCC, these results confirm that taeniasis and cysticercosis represent an impactful public health concern in the Bolivian Chaco. In accordance with WHO/PAHO recommendations [24–26], surveillance programs are needed to define whether the *T. solium* life cycle is still active in the surveyed communities of the Bolivian Chaco and whether adequate control programs are required.

## Supporting information

S1 Data This file provides the database reporting the results described and analysed in this study. (XLSX)
